# Coherence in measurement and programming in maternal and newborn health: experience from the BetterBirth trial

**DOI:** 10.1016/j.jclinepi.2019.05.003

**Published:** 2019-09

**Authors:** Natalie Panariello, Amanda Jurczak, Jonathan Spector, Vishwajeet Kumar, Katherine Semrau

**Affiliations:** a Ariadne Labs, 401 Park Drive, Boston, MA 02215, USA; b Global Health, Novartis Institutes for BioMedical Research, 220 Massachusetts Avenue, Cambridge, MA 02139, USA; c Community Empowerment Lab, 26/11 Wazeer Hassan Road, Lucknow 226001, India

**What is new?**

**Key Findings:**
• In this piece, we describe our method of using a composite outcome of maternal and newborn components in our BetterBirth trial to assess mortality and morbidity outcomes of mothers and newborns.

**What this adds to what was known?**
• The most promising interventions working toward the elimination of preventable maternal and neonatal mortality and morbidity link the care of mothers and babies in a way that is both integrated and coordinated; this approach is tied to the widely accepted fact that the health of the mother–newborns dyad is inextricably linked. However, few studies of maternal and newborn health interventions measure and report on both maternal and newborn outcomes. The BetterBirth trial has used the composite measure to preserve this linkage.

**What is the implication and what should change now?**
• The very nature of the composite measure provides a method to quantify and communicate joint impact of care in a way that separating individual maternal and neonatal measures does not.
• Composite outcomes should be used more widely to assess maternal and newborn health interventions.

In research, a composite outcome is a clinically important indicator constructed by combining relevant individual events [1]; study participants experiencing any one of the component events are considered to have experienced the composite endpoint [2]. Although composite outcomes have been used for many years in various fields [1], their application to maternal and newborn health has only recently become more common. Historically, maternal health and newborn health have been siloed disciplines [3], and composite measures in clinical studies have generally been used only for grouping either maternal or neonatal endpoints [4], [5], [6]. However, a paradigm shift toward interconnected care is increasingly a reality in practice, driven by the idea that addressing maternal and neonatal health together is pragmatic, cost-effective, and produces better results [7], [8]. In addition, researchers are beginning to use the measurement benefits of composite outcomes with both maternal and neonatal indicators [9], [10], [11], [12]. We support the use of composite outcomes in the maternal and newborn health field, as they circumvent a contrived prioritization of one-half of the mother–infant pair and acknowledge the interconnectedness of mothers and babies at the time of childbirth. In addition, from a study perspective, we recognize the important measurement and statistical benefits composite measures can provide. Here, we share our experience with using a composite approach in a large maternal–newborn health trial recently completed in Uttar Pradesh, India, to demonstrate the feasibility of use and encourage adoption of this approach when appropriate [13], [14].

The BetterBirth Program assessed the impact of the World Health Organization's (WHO) Safe Childbirth Checklist (SCC) implemented through a peer-coaching program. The SCC is a quality improvement tool comprised 28 items that serve as prompts for health workers to check that essential birth practices have been performed between the time of maternal admission for childbirth and discharge of the mother and baby [15]. It was designed through a unique collaboration of a large, diverse network of practitioners (including nurses, midwives, obstetricians, neonatologists, and pediatricians) with the aim to help health workers reduce maternal and newborn harm, particularly in low- and middle-income countries where rates of avoidable morbidity and mortality are high. Initial testing of an SCC-based quality improvement program at a single center in South India led to substantial improvements in delivery of essential childbirth practices but was not powered to assess health outcomes. The follow-on study, the BetterBirth trial, was a matched-pair, cluster-randomized controlled trial involving 120 facilities in North India that evaluated the impact on provider behaviors and maternal and newborn health outcomes.

The BetterBirth trial presented an opportunity to select an endpoint that reflected the essence of the SCC itself, a tool that was designed to simultaneously improve outcomes for both mothers and newborns. Because the BetterBirth Program aimed to improve maternal *and* newborn health, we naturally sought to incorporate both maternal and neonatal components. Use of a composite was therefore considered from the earliest stages of trial planning. To determine appropriate component measures, we sought expert consultation with clinicians, public health scientists, epidemiologists, and statisticians. Through a Delphi-type process, the group considered frequently used indicators that included stillbirth, neonatal mortality, maternal mortality, and maternal near-miss. Ultimately, the primary outcome was designed to be a composite measure of perinatal mortality (including intrapartum-related stillbirth and neonatal mortality), maternal mortality, and severe maternal morbidity (adapted from WHO near-missed death criteria) within 7 days of birth [14].

In addition to capturing the linkages between mother and infant, the use of the composite outcome is statistically beneficial. Maternal mortality, although unacceptably high, does not occur in Uttar Pradesh at the frequency required to assess clinically important impact, even in our sample size of approximately 170,000 birth events. Incorporating perinatal mortality and maternal morbidity, both of which are associated with much higher event rates, increased the study's statistical efficiency and reduced risk of misclassification. The statistical advantage of incorporating multiple outcomes has also been referenced in studies in developed countries, where severe adverse events are even more rare [12]. In addition, considering that maternal morbidity is a risk factor for maternal mortality, incorporating a marker of severe maternal harm provided at least a rough proxy for maternal mortality. Finally, using a composite mitigated the risk of type I error in analyzing multiple primary outcomes, as we were able to report one outcome with an alpha of 0.05. To supplement the composite, the investigators also analyzed each composite component separately as a secondary outcome [2].

As with any outcome measure, our use of a composite outcome presented its own set of challenges to be considered. Fundamental to the construction of a composite outcome is coherency: the individual components must reflect clinically significant measures of the effect of the specific intervention [1]. This requires investigators to have consensus on the purpose of the intervention and its relevant endpoints. Our expert contributors held varying opinions on assembly of an appropriate composite measure. Although there was a strong consensus regarding maternal mortality and perinatal mortality, the morbidity components prompted debate. Maternal morbidity was deemed valuable to include to supplement maternal mortality rates, but defining its composition required a careful selection process. We ultimately used the WHO near-miss criteria as a basis for the maternal morbidity component, modified to fit our low-resource setting, as many of the original measures were either unrecognizable, not applicable, or unmeasurable in the trial's study sites (rural health facilities). Furthermore, we initially considered incorporating newborn morbidity measures but ultimately did not include them in the composite because it was determined that newborn morbidity rates were not sufficiently well established to confidently assess at baseline, and there was no existing framework to leverage similar to the near-miss criteria for maternal morbidity. Operationally, including newborn morbidity measures was less of a priority for statistical analyses because baseline rates of newborn harm were (tragically) very high solely through the mortality measure.

We also recognized the risk of uncertainty in interpretation of results [16]. Our researchers determined a priori that experiencing any one of the three composite outcome components of maternal mortality, perinatal mortality, and maternal morbidity would constitute a positive composite outcome event (i.e., events were not double-counted in cases where a mother–child pair experienced more than one component). In addition, we averaged the causal effects of the three component outcomes such that each outcome held the same statistical weight in the composite. As an alternative, assigning weights to each component is a viable solution that other maternal and neonatal composites have used [10], [11], [12]. In our analyses, maternal morbidity rates were the main driver of positive composite outcome events because this indicator had both the highest prevalence and most variance. Although our trial showed null results in both the composite outcome as well as in each component when assessed separately, it is possible that the composite shows a statistically different result from individual measures (i.e., statistical insignificance in individual measures despite a significant component measure, or vice versa) [7], [17]. This phenomenon has implications on perception of the efficacy of the intervention in question.

Composite endpoints that incorporate both maternal and newborn outcome measures are increasingly relevant to maternal and newborn health research, as interconnected care continues to rise in clinical practice. The very nature of the composite measure captures the linkage between a mother and her infant and provides a means to quantify and communicate the impact of care that is delivered to the mother–baby pair. A composite outcome met with success in design and conduct of the BetterBirth trial, and we encourage the maternal and newborn health communities to consider this type of endpoint in future studies. We expect that contributions by many stakeholders will help to refine the selection of optimal composite components that are relevant and practical in various contexts and build on experience with analyses and reporting to mitigate potential challenges. We propose that these steps forward in measurement will help to keep pace with integrated strategies now being implemented to achieve better health outcomes for mothers and babies everywhere.

## CRediT authorship contribution statement

**Natalie Panariello:** Conceptualization, Investigation, Writing - original draft, Writing - review & editing. **Amanda Jurczak:** Conceptualization, Investigation, Writing - original draft, Writing - review & editing. **Jonathan Spector:** Conceptualization, Investigation, Writing - original draft, Writing - review & editing. **Vishwajeet Kumar:** Conceptualization, Investigation, Writing - review & editing. **Katherine Semrau:** Conceptualization, Investigation, Writing - original draft, Writing - review & editing, Supervision.
